# Oral frailty subtypes and influencing factors in older patients with ischemic stroke: a latent profile analysis

**DOI:** 10.3389/fpubh.2025.1696147

**Published:** 2025-12-10

**Authors:** Yutong Zhou, Lixia Xie, Wen Zhang, Yao Chen, Pan Li, Yifan Ou, Haoke Shi, Xinhong Yin

**Affiliations:** 1 School of Nursing, University of South China, Hengyang, China; 2 The Second Affiliated Hospital of University of South China, Hengyang, China

**Keywords:** oral frailty, ischemic stroke, older patients, latent profile, influencing factors

## Abstract

**Objective:**

To analyze potential subtypes and influencing factors of oral frailty in older ischemic stroke patients. To support clinical professionals in precisely identifying high-risk populations and to offer a scientific foundation for the creation of tailored and accurate nursing interventions.

**Method:**

A total of 319 older ischemic stroke patients admitted to the Second Affiliated Hospital of the University of South China between December 2024 and February 2025 were selected using a convenience sampling technique. Various assessments were conducted, including the General Information Questionnaire, the Pittsburgh Sleep Quality Index, the National Institutes of Health Stroke Scale, the Geriatric Depression Scale-15, and the Oral Frailty Index-8. Different subtypes of oral frailty were explored using latent profile analysis. Univariate analysis and multinomial logistic regression were performed to investigate the factors influencing the subtypes of oral frailty.

**Results:**

Oral frailty in older ischemic stroke patients can be classified into three categories: oral function decline—high oral frailty group (21.32%); medium in all dimensions—moderate oral frailty group (27.27%); and active social participation—low oral frailty group (51.41%). Multifactorial analysis indicated that age, cognitive impairment, depression, illness recurrence, living alone, albumin, total cholesterol, prothrombin time, and homocysteine are significant factors for moderate and high levels of oral frailty.

**Conclusion:**

This study delineated three distinct subtypes of oral frailty. It is imperative for healthcare professionals to pay particular attention to moderate and high levels of oral frailty in older patients with ischemic stroke. Tailored care plans should be devised for various patient subgroups to enhance their oral health outcomes.

## 1 Introduction

The number of older people is increasing due to society’s rapid aging. The United Nations Population Division projects that the proportion of older adults will double over the next three decades, accounting for 16% to 22% of the world’s population by 2050 (1). This demographic shift will have a significant impact on public health services and socioeconomic development.

Oral frailty is a relatively new concept that has garnered increased attention recently. In 2020, oral frailty was defined clearly by the Japanese Dental Association as a broad range of oral conditions (such as tooth count, oral hygiene, and oral functioning, among others) that are linked to aging and are accompanied by a decline in interest in oral health as well as a decline in the capacity of physical and psychological reserves, among other phenomena and processes of change (2). This definition emphasized the robust correlation between oral decline and overall frailty, implying that oral health issues may serve as an early indicator of poor health in senior adults.

The current research on oral frailty primarily focuses on the prevalence and influencing factors of oral frailty among aged individuals in nursing homes and the community. However, there is still a lack of research on the level of oral frailty and its influencing factors in patients with specific diseases (3–7). Stroke is a prominent cause of death worldwide, characterized by its high morbidity, recurrence, mortality, disability, and economic burden (8). Ischemic stroke (IS) is the most common type of stroke, accounting for approximately 60–80% of all strokes (9). Research has demonstrated a robust bidirectional relationship between ischemic stroke and oral frailty (10). On the one hand, ischemic stroke frequently causes decreased or even paralyzed function of the tongue, pharynx, palate, and masticatory muscles, affecting the capacity to chew and grind food, resulting in dysphagia and a decline in oral cavity cleaning ability. Such pathological changes tend to degrade the physiological function and hygienic environment of the oral cavity, thereby raising the risk of oral frailty (9, 11). On the other hand, oral frailty has a considerable negative impact on the recovery of ischemic stroke patients. Oral frailty can cause eating disorders, resulting in inadequate nutrient intake, weakening the patient’s immune system, and slowing recovery through processes including chronic inflammation (12). Additionally, oral frailty may have a severe impact on the patient’s psychological health, resulting in lower self-esteem, social activities, and drive for recovery (13). Consequently, it is imperative to monitor the degree of oral frailty in older patients with ischemic stroke and identify the pertinent influencing factors to enhance their quality of life and facilitate their rehabilitation.

However, both domestically and globally, research on oral frailty in older ischemic stroke patients is still in its infancy. Current approaches employ variable-centered methods such as logistic regression and cluster analysis. Despite their value, these techniques have serious drawbacks when it comes to assessing oral frailty: Traditional clustering methods, while exploratory, primarily rely on descriptive segmentation and lack model-based statistical metrics to objectively determine optimal category numbers. Regression models, on the other hand, emphasize net effects of variables at the aggregate level, frequently ignoring heterogeneous subtypes within patients formed by combinations of multiple characteristics. This paper presents latent profile analysis (LPA) as a model-driven, individual-centered solution to this problem. Its benefits are evident in two ways: First, LPA overcomes the drawbacks of “single-dimensional” techniques by detecting latent, discrete subtypes in the data based on response patterns across many variables, exposing inherent population heterogeneity. Second, it makes it possible to compare and calculate profile numbers using objective fit indices, improving the classification process’s statistical rigor and reproducibility while successfully minimizing the influence of subjective judgment (14). Thus, LPA offers a systematic, comprehensive, and precise approach to investigating the factors that influence the level of oral frailty in older patients with ischemic stroke. This study not only identifies potential subgroup characteristics of oral frailty in this patient population but also provides a scientific foundation for the development of personalized and targeted nursing interventions that can effectively improve patients’ oral health status, enhance their overall quality of life, and expedite the recovery process.

## 2 Materials and methods

### 2.1 Ethics approval

This cross-sectional observational study adhered to the principles outlined in the Declaration of Helsinki. Approval was granted by the Ethics Committee of the University of South China (approval number: HLSC040). Informed consent was obtained from all patients.

### 2.2 Subjects

Convenience sampling was used to choose older persons with ischemic stroke who attended Nanhua University’s Second Affiliated Hospital between December 2024 and February 2025.

Inclusion criteria: (1) age ≥ 60 years old; (2) ischemic stroke patients diagnosed by cranial MRI or CT; (3) stable condition, normal cognition, and ability to communicate in normal language; (4) patient and family informed consent.

Exclusion criteria: (1) combined with traumatic brain injury and other non-cerebrovascular pathologies; (2) people with mental disorders; (3) people with fuzzy consciousness, unable to cooperate with the study; (4) people with clinically diagnosed malignant diseases or patients in the terminal stage of the disease; (5) patients participating in other studies; (6) people who do not want to participate in the study.

### 2.3 Sample size calculation

The sample size was calculated using the cross-sectional study formula: $n=\frac{Z^{2}\times P\times (1-P)}{\delta^{2}}$ . The confidence level was set at 95% (*Z* = 1.96). The study found that the prevalence of oral frailty among older ischemic stroke patients was 63.5%, with a permissible error (*δ*) of 10% of the prevalence rate, equivalent to 0.0635. This led to a necessary sample size of 221. This resulted in a sample size of 221. This investigation necessitated a minimum of 266 patients, as 20% of the questionnaires were invalid.

### 2.4 Formulation of influencing factors for the subtypes of oral frailty

Based on the expert meeting’s findings and the literature, a total of 25 factors that influence the subtypes of oral frailty in older ischemic stroke patients were identified, including:

(1) General demographic factors (9 items): age, sex, body mass index (BMI), education, marital status, income, form of residence, smoking history, and drinking history.
(2) Clinical disease-related factors (6 items): neurological function, number of medications, disease condition, cognitive function, sleep status, and depression.
(3) Laboratory test indicators (10 items): platelets, hemoglobin, albumin, total cholesterol, triglycerides, low-density lipoprotein, uric acid, homocysteine (Hcy), C-reactive protein, and prothrombin time (PT).

### 2.5 Survey tools

(1) General Information Questionnaire: The researcher developed a questionnaire. The following factors were considered: age, sex, BMI, education, marital status, monthly per capita family income, smoking history, drinking history, form of residence, disease condition, and number of medications.
(2) Oral Frailty Index-8 (OFI-8) (15): This scale was created by Tanaka et al. through expert consultation and is appropriate for the rapid screening of patients at a high risk of oral frailty. Eight items comprise the scale: (1) Whether it is more challenging to consume hard foods than it was 6 months ago; (2) Whether one occasionally chokes on tea or soup; (3) Whether one wears dentures; (4) Whether one experiences dry mouth; (5) Whether one has been out less than 6 months; (6) Whether one is capable of chewing hard foods, such as peanuts or pickled carrots; (7) Whether one brushes at least twice a day; (8) Whether they visit the dentist at least once a year. The scale had a total Cronbach’s *α* coefficient of 0.692, and a score of 4 or higher was considered indicative of oral infirmity. The score ranged from 0 to 11.
(3) National Institutes of Health Stroke Scale (NIHSS) (16): The scale is used in clinical practice to evaluate the neurological deficit status of patients with acute stroke. It is a comprehensive physical assessment of the patient’s condition, evaluating 11 key aspects, including consciousness, visual field, facial paralysis, limb movement, sensation, speech, and vision. The total score on the scale ranges from 0 to 42, with higher scores indicating more severe neurological impairments. Patients with a baseline assessment of >16 points were at a high risk of death, while those with a baseline assessment of <6 points were more likely to recover. The Cronbach’s a-value of this scale was 0.886, indicating a high level of reliability and validity.
(4) Pittsburgh Sleep Quality Index (PSQI) (17): The scale was created by Buysse, a sleep specialist at the University of Pittsburgh, to evaluate a patient’s sleep over the past month. The PSQI comprises 18 items that cover 7 dimensions: self-perceived sleep quality, time to sleep, total sleep time, sleep efficiency, sleep disorder status, hypnotic drug use, and daytime dysfunction. Each component is scored from 0 to 3 points, with four distinct levels. The cumulative total of each entry is the total PSQI score, which ranges from 0 to 21 points. Higher scores indicate a poorer quality of sleep, while a PSQI score of more than 7 points is used to diagnose sleep disorders. The Cronbach’s *α* coefficient was 0.796.
(5) Geriatric Depression Scale-15 (GDS-15) (18): Sheikh et al. simplified the 30-item GDS scale created by Brink et al. The scale consists of 15 items that cover the fundamental manifestations of depression in older adults, and participants answered “yes/no” to each question based on the statement, with “yes” receiving one point and “no” scoring zero points. The first, fifth, seventh, and eleventh entries were scored 1 point each, resulting in a total score of 0–15 points. Higher scores indicate more obvious depression symptoms, whereas ≥8 points indicate the existence of depressed symptoms. Woo J and Mui Jinrong assessed the scale’s reliability, and the sensitivity was 0.96, specificity 0.88, and internal consistency 0.9. The Cronbach’s *α* coefficient was 0.82, indicating that the measure is valid for assessing depression in older persons.
(6) Mini-Mental State Examination(MMSE) (19): The MMSE, which was created by American scholars Folstein et al., is the most frequently employed cognitive function screening instrument worldwide. The MMSE is a comprehensive evaluation of 11 cognitive functions, including spatial–temporal orientation, short-term memory, attention and computation, recall, and language. A total score of 30 points is required to diagnose cognitive dysfunction, with a score of less than 24 points indicating more severe cognitive impairment in the corresponding domains. The cognitive impairment in the corresponding domain is more severe when the score is lower. The retest reliability of the Chinese version of the scale was 0.924, and Cronbach’s *α* coefficient was 0.833.

### 2.6 Survey and data collection methods

The data collectors received unified training before the survey, which included the main points and precautions of the questionnaire, as well as communication skills for interacting with patients. Before distributing the questionnaire to patients, the investigator asked them to verify their identity, explain the primary content of the questionnaire, and complete the notes. For participants who were unable to independently complete the questionnaire, members of the research team meticulously guided them through each item, ensuring clarity and accuracy by repeatedly confirming their responses before completing the form on their behalf. The questionnaires were collected on-site and immediately examined for completeness and validity upon retrieval. Subsequently, they underwent a dual verification process, during which any incomplete responses were excluded from the analysis. A total of 330 questionnaires were distributed, and 319 valid questionnaires were recovered, resulting in an effective recovery rate of 96.7%.

### 2.7 Statistical analysis

Mplus 8.0 and SPSS 26.0 were employed to analyze the data. The number of categories in the model was gradually increased until the model fit indicators were optimized, and the four-dimensional scores of OFI-8 were used as exogenous variables. This work uses specific algorithmic settings in latent profile analysis to guarantee the precision and stability of model estimation: the maximum iteration count is set at 5,000 to give sufficient search space for model fitting; The convergence tolerance used the default standard of 0.0000001, with convergence determined when the change in log-likelihood between consecutive iterations went below this threshold. Each model also used 100 random beginning values and performed 20 final-stage optimizations to prevent local optima. The optimal log-likelihood values were duplicated by all successfully converged models, demonstrating the excellent stability and dependability of the solutions. The evaluation was conducted using the following metrics: (1) Akaike information criteria (AIC), Bayesian information criteria (BIC), and sample-size-adjusted BIC (aBIC). A model with lower values is considered more effective. (2) The higher the entropy value, the greater the classification accuracy; typically, an entropy value greater than 0.8 is considered a benchmark for high classification accuracy. (3) The Lo–Mendell–Rubin likelihood ratio test (LMR) and the bootstrap likelihood ratio test (BLRT). The k-category model was found to be superior to the k-1 category model, as evidenced by the substantial level of differences in fit between the two models using LMR and BLRT. The *χ*^2^ test or one-way ANOVA was employed to compare the factors that affect older patients with ischemic stroke who have varying levels of oral frailty. The independent variables were factors that were statistically distinct in the one-way analysis of variance, while the dependent variables were the results of the latent category analysis. The influencing factors were analyzed using multinomial logistic regression. The two-tailed test level is *α* = 0.05.

## 3 Results

### 3.1 General information and oral frailty scores

This study obtained a total of 319 valid questionnaires, with a mean age of 74.20 ± 8.47 years and a range of 60–95 years for the participants. The mean oral frailty score of older patients with ischemic stroke was 4.62 ± 3.03, with a range of 0–11.

### 3.2 Subtypes of oral frailty in older ischemic stroke patients and nomenclature

Tanaka classified the OFI-8 scale entries into four categories: poor oral function (entries 1–4), tooth loss (entry 5), declining social participation (entry 6), and oral health-related behaviors (entries 7–8) (4). The four dimensions’ scores were used as exogenous variables in this investigation, and a step-by-step process was used to construct 1–5 possible category models for the prospective profile analysis of oral frailty in older ischemic stroke patients. Models from Class 4 and Class 5 were eliminated based on the LMR criterion of *p* < 0.05. Entropy values exceeded 0.8 in models with one to three potential profiles, and as the number of profiles increased, so did the AIC, BIC, and aBIC values. When the properties of the three likely classes were combined, the best model fit was discovered. The three latent patterns of oral frailty that we finally decided to categorize are shown in Table 1.

**Table 1:** Comparison of oral frailty latent profile analysis indicators in older patients with ischemic stroke.

Model	AIC	BIC	aBIC	Entropy	*P*		Class probability
					LMR	BLRT	
1	3409.063	3439.185	3413.810				
2	3191.203	3240.151	3198.917	0.861	<0.001	<0.001	0.555/0.445
3	3171.328	3239.102	3182.009	0.828	0.004	<0.001	0.514/0.273/0.213
4	3148.022	3234.621	3161.670	0.909	0.058	<0.001	0.179/0.348/0.398/0.075
5	3157.800	3263.226	3174.415	0.906	0.734	1	0.000/0.357/0.398/0.179/0.066

AIC, Akaike information criterion; BIC, Bayesian information criterion; aBIC, sample-corrected BIC; LMR, LoMendell-Rubin; BLRT, Bootstrap-based likelihood ratio test.

**Figure 1:**
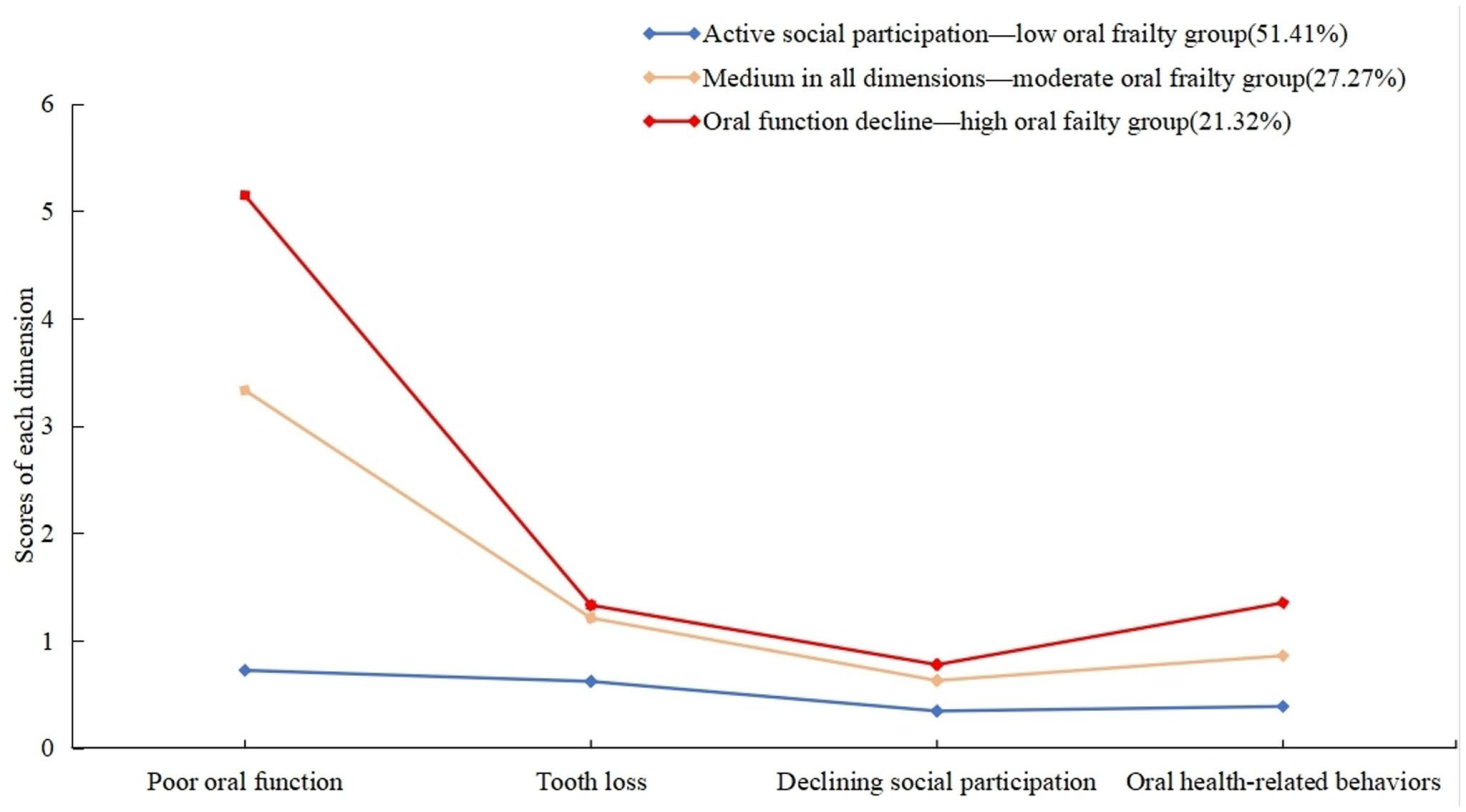
Scores of oral frailty in 3 categories in older patients with ischemic stroke.

The “Oral function decline—high oral frailty group” was named after the fact that older patients with ischemic stroke in category 1 had higher scores in all dimensions of oral frailty, with the highest score on “poor oral function.” The “Medium in all dimensions—moderate oral frailty group” was established for older patients with ischemic stroke who were classified as Category 2. These patients exhibited moderate scores in all dimensions of oral frailty. The “Active social participation—low oral frailty group” was named after Category 3 older patients, who had fewer scores in all dimensions of oral frailty, with the lowest score on “Declining social participation.” (Illustrated in Figure 1).

### 3.3 Univariate analysis of oral frailty subtypes in different older patients with ischemic stroke

Univariate analysis revealed statistically significant differences among the three categories of oral frailty in older ischemic stroke patients regarding age, sex, living alone, illness recurrence, cognitive impairment, depression, and certain laboratory indicators, *p* < 0.05 (Table 2).

**Table 2:** Influencing factors for the three latent oral frailty profiles in older patients with ischemic stroke (*n* = 319).

Variables	C1 (*n* = 68)	C2(*n* = 87)	C3 (*n* = 164)	χ^2^/*F*	*P*
Sex				35.203	<0.05
Male	19	47	115		
Female	49	40	49		
BMI (kg/cm^2^)				4.574	0.334
<18.5	19	26	54		
18.5~	23	25	62		
24~	26	36	48		
Degree				3.214	0.782
Primary school and below	10	10	13		
junior high school	19	23	49		
High school or junior college	25	32	66		
College and above	14	22	36		
Marital status				4.191	0.123
Married	50	75	127		
Unmarried/divorced/widowed	18	12	37		
Living alone				35.510	<0.05
Yes	55	49	63		
No	13	38	101		
Smoking history				0.611	0.737
Yes	35	48	82		
No	33	39	82		
Drinking history				4.122	0.127
Yes	22	28	71		
No	46	59	93		
Monthly per capita family income (yuan)				6.291	0.391
<2,000	9	11	14		
2,000~	22	20	61		
5,000~	30	47	79		
>10,000	7	9	13		
Neurological function				11.610	0.071
Mild stroke	25	36	74		
Moderate stroke	17	22	57		
Moderate to severe stroke	14	16	22		
Severe stroke	12	13	11		
Disease condition				9.125	<0.05
Disease premiere	25	26	31		
Disease recurrence	43	61	133		
Number of medications				43.315	<0.05
≤4 kinds	24	52	131		
≥5 kinds	44	35	33		
Cognitive impairment				7.593	<0.05
Yes	15	37	62		
No	53	50	102		
Sleep disorder				4.625	0.099
Yes	19	36	70		
No	49	51	94		
Depression				41.869	<0.05
Yes	26	21	8		
No	42	65	157		
Age	78.735 ± 8.449	76.126 ± 8.673	72.750 ± 8.310	13.256^a^	<0.05
Platelet	225.515 ± 39.106	222.471 ± 34.604	223.177 ± 38.787	0.135^a^	0.874
Haemoglobin	119.631 ± 7.242	120.097 ± 7.095	120.025 ± 6.381	0.106^a^	0.900
Albumin	40.629 ± 5.045	40.824 ± 3.992	43.9476 ± 4.770	19.142^a^	<0.05
Total cholesterol	6.242 ± 0.967	5.444 ± 0.529	5.411 ± 0.040	44.066^a^	<0.05
Triglycerides	2.174 ± 0.769	2.188 ± 0.850	2.041 ± 0.876	1.104^a^	0.333
Prothrombin time	9.872 ± 0.742	9.779 ± 0.463	9.312 ± 0.292	47.836^a^	<0.05
Low-density lipoprotein	3.679 ± 0.519	2.648 ± 0.552	2.490 ± 0.599	3.675^a^	<0.05
Uric acid	436.103 ± 39.167	426.529 ± 49.434	425.116 ± 50.715	1.297^a^	0.275
C-reactive protein	8.297 ± 5.148	8.651 ± 4.477	8.765 ± 3.885	0.280^a^	0.756
Homocysteine	18.129 ± 2.068	16.792 ± 2.327	16.457 ± 2.412	12.629^a^	<0.05

BMI, Body Mass Index; ^a^F; C1, Oral function decline-high oral frailty group; C2, Medium in all dimensions-moderate oral frailty group; C3, Active social participation-low oral frailty group.

### 3.4 Multinomial logistic regression of oral frailty subtypes in older patients with ischemic stroke

Continuous variables with statistical significance in univariate analysis were normalized to remove dimensional discrepancies across variables and improve data comparability. The presence of multicollinearity was evaluated using “regression model tolerance” and “Variance Inflation Factor (VIF)” before performing unordered multinomial logistic regression. Table 3 displays the results. Every variable showed tolerances >0.1 and VIF values <10, suggesting that there was no discernible multicollinearity between them.

**Table 3:** Results of multicollinearity tests.

Variables	VIF	Regression model tolerance
Age	1.064	0.940
Sex	5.819	0.172
Disease condition	7.413	0.135
Number of medications	9.115	0.110
Cognitive impairment	2.133	0.469
Depression	1.796	0.557
Albumin	1.099	0.910
Total cholesterol	1.110	0.901
Prothrombin time	1.194	0.837
Low-density lipoprotein	1.037	0.964
Homocysteine	1.080	0.926

VIF, Variance Inflation Factor.

Therefore, this study employed the three latent categories of oral frailty as dependent variables. Using the “Active social participation-low oral frailty group” as the reference category, it conducted multinomial logistic regression analysis with variables that demonstrated statistical significance in univariate analysis as independent variables (Table 4).

**Table 4:** Multinomial logistic regression of the three latent oral frailty profiles in older patients with ischemic stroke (*n* = 319).

Project	*β*	SE	Wald *χ*^2^	*P*	*OR*	95%*CI*
C1 vs. C3						
Age	0.484	0.212	5.227	0.022	1.623	1.072–2.457
Living alone						
Yes	2.086	1.035	4.064	0.044	8.051	1.060–61.172
Cognitive impairment						
Yes	2.050	0.918	4.991	0.025	7.770	1.286–46.947
Depression						
Yes	1.873	0.686	7.466	0.006	6.509	1.698–24.951
Albumin	−0.617	0.236	6.865	0.009	0.540	0.340–0.856
Total cholesterol	1.204	0.248	23.534	<0.001	3.334	2.050–5.423
homocysteine	0.559	0.213	6.878	0.009	1.748	1.152–2.654
Prothrombin time	1.220	0.263	21.583	<0.001	3.388	2.025–5.670
C2 vs. C3						
Age	0.375	0.169	4.929	0.026	1.455	1.045–2.027
Disease condition						
Disease recurrence	2.294	0.992	5.344	0.021	9.913	1.418–69.318
Cognitive impairment						
Yes	1.841	0.565	7.869	0.005	6.304	1.742–22.820
Depression						
Yes	2.289	0.696	10.806	0.001	9.868	2.520–38.637
Albumin	−0.623	0.187	11.096	0.001	0.537	0.372–0.774
Prothrombin time	1.323	0.236	31.614	<0.001	3.756	2.363–5.970

C1, Oral function decline-high oral frailty group; C2, Medium in all dimensions-moderate oral frailty group; C3, Active social participation-low oral frailty group.

The results showed that age (OR = 1.623, *p* = 0.022, 95% CI: 1.072–2.457), cognitive impairment (OR = 7.770, *p* = 0.025, 95% CI: 1.286–46.947), living alone (OR = 8.051, *p* = 0.044, 95% CI: 1.060–61.172), depression (OR = 6.509, *p* = 0.006, 95% CI: 1.698–24.951), albumin (OR = 0.540, *p* = 0.009, 95% CI: 0.340–0.856), total cholesterol (OR = 3.334, *p* < 0.001, 95% CI: 2.050–5.423), prothrombin time (OR = 3.388, p < 0.001, 95% CI: 2.025–5.670), and homocysteine (OR = 1.748, p = 0.009, 95%CI: 1.152–2.654) were the factors influencing the patients with “Oral function decline—high oral frailty group.” The factors that influenced patients in the “Medium in all dimensions—moderate oral frailty group” were as follows: age (OR = 1.455, *p* = 0.026, 95%CI: 1.045–2.027), cognitive impairment (OR = 6.304, *p* = 0.005, 95%CI: 1.742–22.820), depression (OR = 9.868, *p* = 0.001, 95%CI: 2.520–38.637), disease recurrence (OR = 9.913, *p* = 0.021, 95%CI: 1.418–69.318), albumin (OR = 0.537, *p* = 0.001, 95%CI: 0.372–0.774), and prothrombin time (OR = 3.756, *p* < 0.001, 95%CI: 2.363–5.970).

## 4 Discussion

Oral frailty in older patients with ischemic stroke can be classified into three categories. Group 1 older ischemic stroke patients exhibit high scores across all oral frailty dimensions, with the highest score in “Poor oral function,” termed the “Oral function decline—high oral frailty group,” accounting for 21.32%. The second category of older ischemic stroke patients exhibits moderate scores across all oral frailty dimensions, termed the “Medium in all dimensions—moderate oral frailty group,” accounting for 27.27%. The third category of older patients demonstrates low scores across all oral frailty dimensions, with the lowest score in “Declining social participation,” termed the “Active social participation—low oral frailty group,” comprising 51.41%.

### 4.1 Three latent profiles of oral frailty in older ischemic stroke patients

Severe oral frailty was observed in 21.32% of older ischemic stroke patients in this study. It has been pointed out that ischemic stroke may cause damage to the neurological pathways innervating the swallowing muscles in older persons and induce the loss of the muscle strength connected to mastication, which results in the decrease of the patient’s oral function (9, 11). Severe deterioration of oral function may increase the risk of infection, which is detrimental to the patient’s recovery. Nursing interventions should concentrate on neuromuscular function rehabilitation in light of this subtype’s characteristics. Precise preventive care programs should be created to lower the likelihood of problems by incorporating specific swallowing exercises and masticatory muscle rehabilitation training. Additionally, 27.27% of patients exhibited moderate oral fragility. Ischemic stroke frequently causes some degree of motor impairment, and patients often struggle to recover effectively. This makes it difficult for individuals to participate in social activities, and the reduction in social interactions can hinder patients’ access to important medical resources and information, negatively impacting their oral health (20, 21). In order to combat the detrimental consequences of functional limits and social isolation, nursing interventions for this subtype should concentrate on reestablishing social functioning through the creation of tiered social activities and information support programs. Finally, 51.41% of patients had a lower level of oral frailty, likely due to the disease having a less significant impact on their oral function. They often practice good oral hygiene, are socially active, and can do routine oral cleaning and maintenance to maintain oral health. This subtype’s traits serve as a guide for creating consistent health maintenance programs. The strategic benefit of tailored prevention is demonstrated by the care’s emphasis on preventing oral functional deterioration through improved social interaction and support for healthy behaviors.

### 4.2 Factors influencing the subtypes of oral frailty in older ischemic stroke patients

According to this study, the factors influencing the latent profile of oral frailty were categorized into three groups: general demographic characteristics, disease-related clinical factors, and laboratory test indicators. This has far-reaching implications for enhancing oral health management strategies and optimizing overall therapy for ischemic stroke patients.

#### 4.2.1 General demographic factors

In this study, age and living alone were identified as risk factors for moderate and high levels of oral frailty in older ischemic stroke patients. Numerous international research have shown that psychological disorders and social isolation negatively impact older adults’ dental health and capacity to retain their functioning abilities.

Age has been identified as a risk factor for oral frailty in a meta-analysis, with the prevalence of oral frailty increasing with age (22). Salivary secretion generally diminishes with age in older persons, as does gingival recession, eventual exposure of tooth roots, and degeneration of dental nerves, resulting in decreased oral self-purification and oral resistance (23). Furthermore, aging causes weakened alkaline phosphatase activity in periodontal membrane cells, decreased regenerative capacity and osteogenic activity in periodontal membrane stem cells, physiological gingival recession, demineralization, and softening of dental bone in older adults, all of which contribute to the development of periodontitis, dental caries, and other diseases, as well as oral frailty (24).

Patients who live alone lack the required care and monitoring, making it difficult for them to receive timely oral health care. Simultaneously, people are more likely to suffer from negative emotions like loneliness and depression due to a lack of psychological support and social interaction (25), which can hinder their ability to take care of themselves and reduce how often they practice dental hygiene. A study found that older individuals who frequently ate alone were substantially more likely to develop oral disease than those who ate with others, highlighting the positive impact of social interaction on oral health (23). Furthermore, people living alone often face financial challenges in accessing dental care and are unable to receive timely oral examinations and treatments. Financial difficulty can also contribute to a deterioration in dietary quality and insufficient nutrient intake, weakening oral tissue health and increasing the risk of oral frailty (26).

#### 4.2.2 Clinical disease-related factors

Moderate and high levels of oral frailty are significantly influenced by cognitive impairment, depression, and illness recurrence.

This study demonstrates that cognitive decline is a risk factor for oral frailty on its own. This result is consistent with several worldwide research that found a substantial correlation between oral self-care abilities and post-stroke cognitive impairment. The cognitive impairment that results from brain tissue ischemia and hypoxia caused by ischemic stroke is further exacerbated by inflammatory responses and oxidative stress, which damage neuronal cells (27). The daily behavioral abilities of cognitively impaired patients are correspondingly lower, and they are unable to independently complete oral hygiene and care in the same manner as normal individuals. This can readily lead to oral diseases and, ultimately, oral frailty (28). Furthermore, the gradual accumulation and deterioration of oral issues may occur as a consequence of these patients’ failure to prioritize the hygiene and health of their oral cavity. This exacerbates the risk of oral frailty (29).

Ischemic stroke can disrupt the brain’s systems that regulate mood, particularly neurotransmitters that are closely associated with depressive symptoms, such as 5-hydroxytryptamine (5-HT), norepinephrine, and dopamine. Following a stroke, patients may experience depression as a consequence of the numerous stressors they encounter, such as diminished self-care and different social responsibilities (30). The risk of oral disease is increased in depressed individuals due to their incapacity and indifference to maintaining proper oral hygiene habits. Additionally, xerostomia, which worsens oral infirmity by reducing the mouth’s capacity to self-clean, is commonly brought on by depressive drugs. Because depression affects the neuroendocrine system and causes persistent inflammation, it may also make oral tissue damage worse. Depression significantly increases the risk of oral frailty in older patients with ischemic stroke as a result of a combination of mechanisms (31).

Oral frailty is significantly increased in older individuals who have suffered recurrent ischemic strokes. The accumulation of brain tissue injury in response to recurrent strokes affects the brain’s regulation of oral motor function, thereby weakening the self-cleaning effect of the oral cavity and increasing the risk of oral disease. A harmful loop of oral inflammation and tissue damage can result from the systemic inflammatory response and oxidative stress brought on by repetitive strokes, which can also have an impact on the health of the oral mucosa and periodontal tissues. When these characteristics come together, recurrent stroke patients are more vulnerable to oral insufficiency (32).

#### 4.2.3 Laboratory test indicators

Disturbances in the patient’s internal environment are induced by ischemic stroke through a variety of mechanisms, including immune microenvironmental changes, microcirculatory disturbances, and neuroendocrine disorders. Consequently, certain laboratory indicators may indicate the patient’s oral frailty (11). This work offers fresh scientific data for the investigation of oral frailty. It implies that chronic inflammatory conditions and systemic metabolic problems may be closely linked to oral frailty. Even though there aren’t many studies in the literature yet that specifically look at the relationship between these indicators and oral frailty, this creates a promising new route for future research into the mechanisms of comorbidity between oral frailty and systemic health.

The findings of this investigation revealed that albumin is a protective factor for patients’ oral frailty. Albumin boosts patients’ immunity and tissue healing ability, minimizes brain tissue damage from ischemia, protects the oral mucosa, and lowers the risk of mouth dryness and infection. Furthermore, the antioxidant capabilities of albumin can attenuate oxidative stress damage to oral tissues, thereby reducing the incidence of oral frailty (33).

This study discovered that longer prothrombin time was associated with oral frailty. Prolonged PT is a sign of coagulopathy, which raises the risk of oral bleeding, slows wound healing, and can lead to infection, exacerbating oral weakness. Anticoagulation (e.g., warfarin) may prevent stroke recurrence; nevertheless, it can prolong PT and increase the risk of bleeding (34). Long-term PT prolongation may also be related to malnutrition, which impairs the function of the oral mucosa and salivary glands, resulting in dry and fragile mucous membranes, decreased saliva output, impaired oral self-cleaning ability, and an increased risk of infection. These variables combine to form a vicious cycle that exacerbates oral health issues.

High levels of homocysteine (Hhcy) are closely associated with vascular endothelial dysfunction, which can lead to atherosclerosis and affect the blood supply to oral tissues. Additionally, HHcy can induce oxidative stress, which can damage the oral mucosa and periodontal tissues, thereby increasing the risk of oral disease. Oxidative stress is also induced by HHcy, which increases the risk of oral diseases and damages the oral mucosa and periodontal tissues. Furthermore, HHcy is frequently associated with deficiencies in vitamin B12, B6, and folic acid, which are crucial for maintaining oral mucosal health. The oral mucosa’s capacity to regenerate itself is impaired, which further increases the risk of oral frailty (35).

Older ischemic stroke patients with elevated cholesterol are also more prone to developing oral fragility. Atherosclerosis narrows blood vessels and reduces blood flow, affecting the blood supply and nutritional status of oral tissues. Inflammatory responses and oxidative stress caused by high cholesterol can further damage the oral mucosa and periodontal tissues, increasing the risk of oral disease (36). In addition, lipid-lowering medications may induce adverse effects, including xerostomia, which impairs oral self-cleaning capacity and exacerbates oral frailty.

In conclusion, healthcare professionals should carry out comprehensive oral training for older patients with ischemic stroke, including oral functional exercise and continuity training, to enhance oral function; establish multidisciplinary teamwork, with nursing staff, dietitians, oral specialists, and other professionals working together to formulate personalized care plans, provide nutritional support, psychological care, and oral health education; optimize the care regular assessment and feedback can ensure the effectiveness of preventive measures and timely adjustments to care strategies, as well as rational medication management to reduce side effects; encourage patients to participate in social activities to reduce the sense of isolation caused by living alone; and, at the same time, improve the living environment, increase public activity space, and promote social participation. Nursing staff can significantly minimize oral frailty in older ischemic stroke patients and improve their transition to health by using a complete set of medical, psychological, and social support strategies.

## 5 Limitations and prospects

This study has several limitations. The sample representation was restricted to Hengyang and lacked validation at various times and locations. This study employed convenience sampling, which may introduce selection bias and affect the generalizability of the results. The cross-sectional survey design was employed, which limited the data to a specific point in time and prevented a comprehensive assessment of the direct effect between factors. Additionally, the absence of patient follow-up rendered it impossible to evaluate trends in oral frailty over time and the long-term development of patients.

Future research should use probability sampling to choose patient cohorts that span a range of time periods and geographical locations in order to improve the representativeness and generalizability of study findings. Additionally, we will evaluate the temporal trends of oral frailty over time as well as the long-term progression of patients using a longitudinal study design that includes patient follow-up. In order to better understand the dynamic linkages and underlying mechanisms among these factors, it will be possible to clarify the time sequence and causal direction between oral frailty and recognized factors (such as living alone, cognitive impairment, biomarkers, etc.).

## 6 Conclusion

The latent profile analysis (LPA) classified oral frailty in older patients with ischemic stroke into three subtypes: Oral function decline—high oral frailty group (21.32%), medium in all dimensions—moderate oral frailty group (27.27%), and active social participation—low oral frailty group (51.41%). Age, cognitive impairment, depression, albumin, prothrombin time, cholesterol, homocysteine, multiple episodes, and living alone all significantly influenced the subtypes. Healthcare professionals can enhance patients’ oral health and overall quality of life by creating more precise interventions tailored to the patient’s subtypes of oral frailty and the associated influencing factors.

## Data Availability

The raw data supporting the conclusions of this article will be made available by the authors, without undue reservation.
